# Dr. Ambler Tees, Sr.

**Published:** 1891-05

**Authors:** 


### DR. AMBLER TEES, SR.

THE sudden death of Dr. Tees in the midst of his active usefulness has created a deep impression. He has always been a worker. Few have devoted as much time and interest as he to the elevation of his favorite calling in this, the city of his birth, and in the country at large.

He was born in Philadelphia, August 20, 1836, and died April 11, 1891, aged fifty-five years.

He graduated at the high-school in Philadelphia at the age of seventeen, and subsequently received the degree of master of arts.

He studied .dentistry in New York with his brother-in-law, Dr. J. G. Ambler, and began the practice in 1856. He matriculated in the Philadelphia Dental College in 1864, and graduated with the first class of that college in 1866. While a resident of New York he studied medicine at the University of the State of New York.

Dr. Tees was an active society member, and became connected with the Odontographic Society of Philadelphia, the Odontological Society of Pennsylvania, and was also an active worker in the American Dental Convention during the closing years of its existence. His long and faithful work as secretary of the Odontological Society had much to do with the success of that organization.

The "Lilliput Furnace" for "Continuous Gum," which bears his name, gave him a national reputation. The "Tees Clamps" are still among the most valuable of these forms of instruments, and his subalveolar forceps have been appreciated by many.

His death was due to angina pectoris and heart complications.

Dr. Tees was a man of intense convictions. His positiveness of character oftentimes led him to be misunderstood, but those who knew him best appreciated his sterling integrity and moral worth. His skill in the preparation of continuous gum and in general prosthetic dentistry has been fully appreciated in Philadelphia, and led to special appointments in the University of Pennsylvania, Department of Dentistry, and in other schools.

The silent reaper is rapidly gathering the fruitage of many years. One after another of the earnest workers are being called away, and the duties they so ably performed must fall to other shoulders. May these be as well and worthily done as by the subject of this sketch.

